# Perinatal outcomes of hypertensive disorders in pregnancy at a tertiary hospital in Ghana

**DOI:** 10.1186/s12884-017-1575-2

**Published:** 2017-11-21

**Authors:** Kwame Adu-Bonsaffoh, Michael Y. Ntumy, Samuel A. Obed, Joseph D. Seffah

**Affiliations:** 1 Department of Obstetrics and Gynecology, Korle Bu Teaching Hospital, Korle Bu, Accra, Ghana; 2 Department of Obstetrics and Gynecology, School of Medicine and Dentistry, University of Ghana, Accra, Ghana; 3 Department of Physiology, School of Allied and Biomedical Science, University of Ghana, Accra, Ghana

**Keywords:** Preeclampsia, Eclampsia, Gestational hypertension, Chronic hypertension, Perinatal outcomes, Ghana

## Abstract

**Background:**

Hypertensive disorders in pregnancy remain a major global health issue not only because of the associated high adverse maternal outcomes but there is a close accompaniment of significant perinatal morbidity and mortality especially in Sub-Saharan Africa (SSA). However, the perinatal burden of HDP in Ghana has not been explored. We conducted this study to determine the perinatal outcomes of HDP at a tertiary hospital in Ghana.

**Methods:**

A cross-sectional study conducted between January to February 2013 at Korle Bu Teaching Hospital (KBTH) in Accra, Ghana. Data collection involved baseline review of all the obstetric population who had just delivered to identify those with HDP. An informed consent was obtained after which a structured questionnaire was adminstered to the hypertensive mothers. The medical records of the mothers and their babies were also reviewed to determine the perinatal outcome indicators of relevance to the study. Data obtained were analyzed using SPSS version 20.

**Results:**

We included 368 women with HDP and singleton births with a mean gestational age at delivery of 37.4 ± 3.3 weeks. Adverse perinatal outcomes determined include the following: 91 (24.7%) neonates were admitted to the Neonatal Intensive Care Unit, 56 (15.2%) had neonatal respiratory distress/asphyxia with 14 (3.8%) requiring ventilatory support and 80 (21.7%) were delivered preterm. Also, stillbirth, early neonatal death, intrauterine growth restriction and low birth weight occurred in 25 (6.8%), 14 (3.8%), 23 (6.1%) and 91 (24.7%) respectively with a perinatal mortality rate of 106 per 1000 births. One and 5 minute APGAR scores <7 occurred in 125 (34.0%) and 55 (14.7%) neonates respectively. Most of the adverse perinatal outcomes were significantly more common in those with preeclampsia compared to the other hypertensive disorders.

**Conclusion:**

There is a significant burden of perinatal morbidity and mortality associated with HDP in the Ghanaian obstetric population and these adverse outcomes were more prevalent in preeclampsia compared to the other hypertensive disorders. Regular goal-oriented clinical audit into perinatal morbidity and mortality associated with HDP and an active multidisciplinary approach to the management of these disorders in the hospital might improve the clinical outcomes of women with maternal hypertension.

## Background

Hypertensive disorders in pregnancy (HDP) remain a major global health issue not only because of the associated high adverse maternal outcomes but there is a close accompaniment of significant perinatal morbidity and mortality [1–3]. Although most obstetricians worry more about the risk of maternal death in women whose pregnancies are complicated by hypertensive disorders the risk of perinatal death is more daunting. For instance, the risk of maternal death is less than 1% in severe preeclampsia and whereas that of perinatal death is about 13%. The situation is even worse in eclampsia where the risks of maternal and perinatal deaths occur in about 5% and 28% respectively [4]. The other side of the coin is the occurrence of serious short and long term complications in the surviving newborns such as the risk of neuro-developmental deficits especially in poorly resourced countries [1].

Generally, there is disproportionately high neonatal mortality in SSA and most of these occur during the first 4 weeks of life although being a newborn is not, in itself, a disease. It is estimated that for every early neonatal death there is another baby that is born dead (stillbirth) and HDP account for most of these perinatal losses especially in low resource settings [5]. The adverse perinatal outcomes associated with hypertensive disorders are generally referable to placental insufficiency, placental abruption and prematurity-related complications [2, 3]. Adverse perinatal outcomes due to HDP or maternal hypertension are generally most severe in severe preeclampsia/eclampsia and are usually dependent on the gestational age at delivery as well as the severity of the disease process.

Perinatal mortality is a key indicator of maternal care and a reflection of the quality of obstetric and pediatric care available [5]. Global perinatal mortality rate is estimated as 47 per total births with excessively wide disparity between the developed (10 per births) and less developed regions such as West Africa (76 per births). In Ghana, the perinatal mortality is estimated as 45 per 1000 deliveries [5].

More recently, the maternal outcomes associated with HDP at KBTH were determined and a high burden of maternal morbidity and mortality were reported [6]. However, the data regarding perinatal outcomes of these heavily prevalent disorders are lacking in our indigenous setting. In KBTH, where the current study was undertaken, there is a general knowledge (based on clinical practice and expert opinion) among the obstetric and pediatric healthcare providers that HDP accounts significantly for a high proportion of perinatal adverse events although scientific documentation of this clinical impression is limited. The objective of the study was to determine the perinatal outcomes of hypertensive disorders in pregnancy among pregnant women obtaining maternity and childbirth services at Korle Bu Teaching Hospital in Accra, Ghana. The findings of this study represent an evidence for healthcare providers and policy makers in devising more appropriate interventions to improving maternal and perinatal health among pregnant women with hypertensive disorders especially in low resource settings.

## Methods

This cross-sectional study was conducted at the Maternity unit of KBTH in Accra between 1st January and 28th February 2013. Korle Bu Teaching Hospital is the largest teaching hospital in Ghana with over 10,000 deliveries annually, and it serves as a tertiary referral centre within a catchment area of about 50 km radius and a population of over 3 million. In this study, we included all women with hypertensive disorders in pregnancy delivering at KBTH who consented to partcipate in the study. Excluded from the study were women with HDP who declined to participate in the study and those with hypertension who delivered in other health facilities before referral to KBTH. Women with twin gestations were also excluded from the study to avoid their potential confounding effect on the adverse perinatal outcomes associated with HDP.

The data collection involved daily identification of all the women who had delivered during the previous 24 h in the hospital. Baseline data were extracted from patients’ folders, admission and discharge registers at the labour wards. After the initial daily baseline data extraction on all the parturient, women with hypertensive disorders were identified and their folder numbers recorded. These women were then assigned study identification numbers after which they were then traced to their respective maternity wards where they had been admitted following delivery. This selected group of women were approached and informed to be included in the study and those who gave written informed consent were included. The purpose of the study was explained to the mothers independently prior to data collection and they were informed that their participation was purely voluntary. The mothers were also informed that failure to participate in or withdraw from the study would not upset the quality of care they would receive for their respective medical conditions.

Their basic socio-demographic characteristics were obtained using a structured questionnaire and their medical records were also reviewed to determine the perinatal outcomes of their pregnancies. The babies of these women (including those admitted to the neonatal intensive care unit-NICU) were followed up on daily basis to find out if they had developed any complication till they were discharged from the hospital. The data obtained from the interviews and the medical records included socio-demographic information (such as age, educational status, marital status) and obstetric data (such as gravidity, parity, gestational age at booking and delivery). The maternal outcomes associated with HDP have recently been published elsewhere [6]. Perinatal outcomes indicators determined included birth weight, neonatal respiratory distress, the need for NICU admission, APGAR scores, stillbirths and neonatal deaths.

In this study, hypertensive disorders in pregnancy were classified as preeclampsia, gestational hypertension, chronic hypertension and preeclampsia superimposed on chronic hypertension [2, 7]. Hypertension in pregnancy was defined as systolic blood pressure (BP) ≥140 mmHg and/or a diastolic BP ≥90 mmHg respectively [2, 3]. Proteinuria was determined using a semi-quantitative dipstick testing. Dipstick proteinuria of ≥1+, in a random urine, was considered significant in the presence of hypertension without evidence of urinary tract infection [3, 7].

We obtained approval for the study protocol from the Ethical and Protocol Review Committee of the School Medicine and Dentistry of the College of Health Sciences, University of Ghana, prior to data collection (Protocol Identification Number: MS-Et/M.11-P.4.9/2011–2012). All the study participants gave a written informed consent prior to the commencement of the study.

The analysis of the data was performed using SPSS version 20.0. Descriptive statistics were performed. The results were presented in percentages where necessary and appropriate measures of centrality (mean) and dispersion (standard deviation) were also calculated. The mean gestational age, systolic and diastolic BP at diagnosis of preeclampsia and gestational hypertension were determined using independent student t-test. Univariate and multivariate analyses were also performed with respect to the occurrence of adverse perinatal outcomes in preeclampsia and the other hypertensive disorders. *P*-values less than 0.05 were considered significant for the differences obtained.

## Results

During the study period, 1856 deliveries were undertaken at KBTH resulting in 1924 total births with 1848 live births. There were 398 women with HDP who delivered over the same period out of which 30 were excluded because 2 declined to give informed consent, 12 had multiple gestations and 16 had incomplete data resulting in a total of 368 cases of HDP which were included in the analysis. Regarding the gestational age at delivery, 288 (78.3%) delivered at ≥37 weeks, 35 (9.5%) between 34 to 36 weeks with 45 (12.2%) of the deliveries occurring below 34 weeks. The mean gestational age at delivery was 37.4 ± 3.3 weeks. Most of the hypertensive mothers [276 (69.9%)] were between 20 to 34 years of age. The maternal socio-demographic and obstetric characteristics are shown in Table 1.

**Table 1:** Socio-demographic and obstetric characteristics of women with hypertensive disorders in pregnancy at KBTH

Variable	Preeclampsia *n* = 140	Gestational Hypertension *n* = 184	Chronic hypertension *n* = 23	Preeclampsia suprimposed on chronic hypertension *n* = 21	Total (*N* = 368)
AGE GROUP < 20 years	9 (6.4)	11 (6.0)	0	0	20 (5.4)
20–34 years	110 (78.6)	122 (66.3)	15 (65.2)	9 (42.9)	276 (69.9)
= > 35 years	21 (15.0)	51 (27.7)	8 (34.8)	12 (57.1)	92 (25.0)
GRAVIDITY 1	57 (40.7)	63 (34.2)	4 (17.4)	6 (28.6)	130 (35.3)
2–4	73 (52.1)	98 (53.3)	17 (73.9)	10 (47.6)	198 (53.8)
= > 5	10 (7.7)	23 (12.5)	2 (8.7)	5 (23.8)	40 (10.9)
PARITY 0	57 (40.7)	63 (34.2)	4 (17.4)	6 (28.6)	130 (35.3)
1–3	73 (52.1)	98 (53.3)	17 (73.9)	10 (47.6)	198 (53.8)
= > 4	10 (7.1)	23 (12.5)	2 (8.7)	5 (23.8)	40 (10.9)
MARITAL STATUS Married/cohabitation	112 (80.0)	145 (78.8)	20 (87.0)	18 (85.7)	295 (80.2)
Single	28 (20.0)	39 (21.2)	3 (13.0)	3 (13.0)	73 (19.8)
EDUCATION None	17 (12.1)	16 (8.7)	3 (13.0)	1 (4.3)	37 (10.1)
Primary school	16 (11.4)	23 (12.5)	5 (21.7)	3 (14.3)	47 (12.8)
Secondary school	91 (65.0)	124 (67.4)	13 (56.5)	14 (66.7)	242 (65.8)
Tertiary	16 (11.4)	21 (11.4)	2 (8.7)	3 (14.3)	42 (11.4)
ANTENATAL VISITS Less than 4	27 (19.3)	22 (12.0)	0	3 (14.3%)	52 (14.1)
4 or more	113 (80.7)	162 (88.0)	23 (100.0)	18 (85.3)	316 (85.9)

Values in the Table are given as number (percentage)

Meconium staining of the liquor occurred in 64 women (17.4%) with the highest (27 women, 19.3%) and lowest (3 women, 13.0%) frequencies occurring in the preeclamptic and uncomplicated chronic hypertensive groups respectively. Cesarean birth occurred in 168 (45.7%) hypertensive mothers. The frequencies of the various perinatal outcome indicators among the different categories of hypertensive disorders are presented in Table 2. Fifty six neonates (15.2%) had respiratory distress or asphyxia which was significantly highest (21.4%) and lowest (4.3%) in preeclamptic and chronic hypertensive groups respectively. The need for ventilatory support occurred in 14 (3.8%) neonates with the highest requirement occurring in the babies of preeclamptic mothers (Table 2).

**Table 2:** Perinatal outcomes among women with hypertensive disorders in pregnancy at KBTH

Perinatal outcome indicators	Preeclampsia n = 140	Gestational Hypertension n = 184	Chronic hypertension n = 23	Preeclampsia suprimposed on chronic hypertension n = 21	Total (N = 368)
Admission to NICU	51 (36.4)	34 (18.5)	2 (8.7)	4 (19.0)	91 (24.7)
Respiratory distress/asphyxia	30 (21.4)	21 (11.4)	1 (4.3)	4 (19.0)	56 (15.2)
Need for ventilatory support	10 (7.1)	3 (1.6)	0	1 (4.8)	14 (3.8)
Stillbirth	13 (9.3)	10 (5.4)	1 (4.3)	1 (4.8)	25 (6.8)
Early neonatal death	8 (5.7)	5 (2.7)	0	1 (4.8)	14 (3.8)
Perinatal death	21 (15.0)	15 (8.2)	1 (4.3)	1 (4.8)	39 (10.6)
Intrauterine growth restriction	15 (10.4)	7 (3.7)	0	1 (4.8)	23 (6.3)
Low birth weight	57 (40.7)	27 (14.7)	0	7 (33.3)	91 (24.7)
Normal birth weight	79 (56.4)	139 (75.5)	18 (78.3)	12 (57.1)	248 (67.4)
Macrosomia	4 (2.9)	18 (9.8)	5 (21.7)	2 (9.5)	29 (7.9)
Preterm delivery	49 (35.0)	25 (13.6)	1 (4.3)	5 (23.)	80 (21.7)
APGAR score < 7 at 1 min	64 (45.7)	51 (27.7)	4(17.4)	6 (28.6)	125 (34.0)
APGAR score < 7 at 5 min	29 (20.7)	22 (12.0)	2 (8.7)	2 (9.5)	55 (14.9)

*IUGR* Intrauterine growth restriction, *LBW* low birth weight, *NICU* Neonatal intensive care unit. Values in the Table are given as number (percentage)

There were clinically significant findings among the types of hypertensive disorders with respect to frequency of NICU admission, neonatal respiratory distress, low birth weight, macrosomia and APGAR score at 1 min. The frequency of low birth weight (LBW) was 24.7% which was highest in the preeclamptic group. On the other hand, macrosomia occurred in 7.9% of the babies which was most frequent in chronic hypertension. Preterm delivery also occurred in 21.7% of the babies with its occurrence being highest (35.0%) and lowest (4.3%) in preeclampsia and chronic hypertension respectively. Regarding APGAR scores, 34.0% and 14.9% of babies had scores of less than 7 at 1 minute and 5 minutes respectively (Table 2).

The occurrence of low birth weight, admission to NICU and low APGAR score after 1 minute of delivery remained significantly higher in preeclampsia compared the other HDP after adjusting for maternal age, parity, number of antenatal visits, gestational age at delivery and the mode of delivery. There was statistically significant difference between preeclampsia and the other HDP regarding birth asphyxia, perinatal deaths and low APGAR after 5 min of delivery in the univariate analysis but the statistical significance disappeared after adjusting for the confounding factors (Table 3). There was a perinatal mortality rate of 106 per 1000 births among women with HDP during the study period.

**Table 3:** Univariate and multivariate analyses of adverse perinatal outcomes in hypertensive disorders in pregnancy at KBTH

Maternal outcome indicators	Preeclampsia n = 140	Other Hypertensive disorders *n* = 228	Unadjusted Odds ratio* (95%CI)	*p* value*	Adjusted Odds ratio** (95%CI)	*P* value**
NICU	51 (36.4%)	40 (17.5%)	2.693 (1.659–4.373)	˂0.001	1.745 (1.007–3.024)	0.047
Low birth weight	57 (40.7)	34 (14.90	3.918 (2.385–6.438)	˂0.001	2.496 (1.272–4.897)	0.008
Stillbirths	13 (9.3%)	12 (5.3%)	1.843 (0.816–4.161)	0.142	1.396 (0.569–3.426)	0.467
Early neonatal deaths	8 (5.7%)	6 (2.6%)	2.242 (0.761–6.605)	0.163	0.807(0.227–2.862)	0.739
Perinatal deaths	21 (15.0%)	18 (7.9%)	2.059(0.435–2.739)	0.037	1.188 (0.542–2.602)	0.667
Birth asphyxia	31 (22.1%)	26 (11.4%)	2.210 (1.248–3.911)	0.006	1.445 (0.760–2.750)	0.262
Low APGAR (1 min)	64 (45.7%)	61 (26.8%)	2.305 (1.480–3.592)	0.001	1.868 (1.118–3.121)	0.017
Low APGAR (5 min)	29 (20.7%)	26 (11.4%)	2.030 (1.139–3.617)	0.023	1.204 (0.603–2.407)	0.599

*Non adjusted *p*-value or Odds ratio, **Adjusted *p*-value or Odds ratio, The perinatal outcome indicators were adjusted for maternal age, parity, number of antenatal visits, gestational age at delivery and mode of delivery. *CI* Confidence interval, *NICU* Neonatal intensive care unit

## Discussion

Hypertensive disorders in pregnancy are associated with significant perinatal morbidity and mortality especially in the developing world. In this study, the major adverse perinatal outcomes determined among women with HDP include intrauterine growth restriction (6.3%), intrauterine fetal death (6.8%), preterm delivery (21.7%), low birth weight (24.7%) and birth asphyxia or neonatal respiratory distress (15.2%) among other complications. We found a perinatal mortality rate of 106 per 1000 births among women with HDP in KBTH. The proportion of perinatal deaths was highest in preeclampsia compared to the other categories with the lowest rate occurring in the chronic hypertensive group. This might be partly attributed to issues related to the quality of care offered to women with HDP as well as complications of prematurity characteristic of these disorders and their management. The high perinatal mortality rate might also be attributed to the tertiary status of KBTH which serves as the referral centre for the primary and secondary health facilities in the southern part of the country. However, this finding agrees with a recent study from a teaching hospital in Nigeria that reported a perinatal mortality rate of 110.3 per 1000 deliveries in hypertensive mothers [8] but lower than the 144 and 317 per 1000-births reported in Turkey and Ethiopia respectively [9, 10].

Similarly, the frequencies of stillbirths (6.8%) and early neonatal deaths (3.8%) were generally high in women having HDP with the highest frequency occurring in the preeclamptic group. It is, however, important to emphasize that there was no early neonatal death and stillbirth in the women with chronic hypertension and superimposed preeclampsia respectively. The finding of significant adverse perinatal outcomes in HDP indicates the importance of optimal antenatal, careful intrapartum and adequate newborn care. This is important because adverse perinatal outcome is a key indicator of maternal health and a reflection of the quality of obstetric and pediatric care [5].

Also, the occurrence of unacceptably high perinatal deaths in HDP indicates the need to revamp the multidisciplinary approach to the management of women with these disorders and their babies in the hospital. A dedicated multidisciplinary team should include active involvement of neonatologist, laboratory staff, midwives, nurses with rich experiences in neonatal care, obstetric physician and obstetricians in the decision making process to ensure effective and comprehensive care for these conditions. This multi-departmental collaboration is indispensable in the provision of evidence based care for women with these conditions especially in SSA where HDP are the leading cause of institutional maternal deaths [11–14]. In KBTH where this study was conducted, HDP are the leading cause of maternal demise responsible for over 30% of such losses and most of these losses are attributable to suboptimal care [11]. The issue of maternal mortality due to HDP should be addressed comprehensively in conjunction with perinatal deaths as the former might predispose to the latter. Recently, Endeshaw and Berhan determined that maternal mortality is an independent risk factor for perinatal death [15] and this suggests that comprehensive approach should be the ultimate in addressing the high maternal and perinatal adverse outcomes characteristic of HDP. Thus, poor perinatal outcomes of HDP may be largely related to suboptimal management of the maternal condition as well as lack of modern neonatal intensive care support for these babies who require specialized clinical care especially in the low resource settings.

The current study showed that the frequency of neonatal respiratory distress (15.2%) and the need for ventilatory support (3.8%) were generally high among the neonates of hypertensive mothers with the highest and lowest rates recorded in the preeclamptic and chronic hypertensive groups respectively. Hauth et al. also reported a similar finding of increased neonatal respiratory distress requiring resuscitation and ventilatory support in babies of hypertensive mothers and these were significantly severe in the preeclampsia [16]. Their study was limited to mild and severe preeclampsia and did not include chronic hypertension and superimposed preeclampsia. However, the current study compared the perinatal outcomes among the various categories of HDP to better understand the relativity of these untoward outcomes.

Other studies have also described similar findings of increased adverse perinatal outcomes in women with hypertensive disorders [17, 18]. Yadav et al. [17] showed that 40% of babies of hypertensive mothers required admission to the NICU and HDP contributed to an estimated 22% of all perinatal deaths. In the current study, NICU admission rate for babies of hypertensive mothers was 24.7% and this was highest and lowest in the women with preeclampsia and chronic hypertension respectively. These adverse perinatal outcomes may be attributed to placental insufficiency, placental abruption and complications of prematurity. The issue of prematurity in relation to HDP is a real disturbing obstacle to improving perinatal survival resulting in major difficulty in deciding the optimal gestational age to deliver these babies. Generally but not surprisingly, the equation is tilted to favour the obstetrician than the neonatologist by resorting to preterm delivery to salvage maternal survival in preference to the fetus with the resultant delivery of babies with varying degrees of intrauterine maturity. Perinatal morbidity and mortality may also result from intrauterine growth restriction which occurred in 6.1% in the current study with the highest rate occurring in women with preeclampsia. This finding is consistent with the incidence of 6.1% and 6.6% reported in Iran and South Africa respectively among pregnant women with hypertensive disorders [18, 19].

There is evidence that most of the maternal deaths associated with HDP are due to substandard care [20]. Similarly, the high prevalence of adverse perinatal outcomes in women with HDP determined in this study might have substandard care as the underlying cause in majority of cases due to clinical management-related challenges in the hospital. We recommend regular and well focused perinatal morbidity and mortality audits in cases of HDP with adverse perinatal outcomes to identify specific areas where significant substandard care contributed to the recorded adversity. Although the optimal management of women with HDP is crippled with myriads of systemic and clinical management-related problems such as lack of up-to-date laboratory support and parenteral antihypertensives in low resource settings, maternal-fetal health may be improved following careful and continuous clinical assessment and timely delivery of the fetus.

Also, the APGAR score of less than 7 at one and 5 minutes occurred in 34.0% and 14.7% respectively in neonates of hypertensive mothers with significantly high proportion of low scores in the preeclamptic group. However, there was substantial improvement in the APGAR scores at 5 min among the various types of hypertensive disorders and this might be partly attributed to a more effective neonatal resuscitation from adequate preparation during the transition to extrauterine life. The finding of significantly low 1 minute APGAR score in babies of hypertensive mothers is consistent with the study by Fatemeh et al. although their study did not include all the women with hypertensive disorders but was limited to primigravidas [18]. Similarly, a recent study in Ethiopia by Wolde et al. also reported low 1 minute APGAR scores in hypertensive disorders with significantly increased frequency in preeclampsia compared to the other categories [10]. These findings suggest the need for adequate preparation for neonatal resuscitation at the time of childbirth in pregnancies complicated by hypertensive disorders.

Regarding birth weight, there were significant differential end points in this study in terms of low birth weight (LBW), normal birth weight and macrosomia occurring in 24.7%, 66.0% and 7.9% respectively. The occurrence of low birth weight was highest in the preeclamptic women and none of the neonates from mothers with uncomplicated chronic hypertension had LBW. Also, the occurrence of macrosomia was significantly highest and lowest in the chronic hypertensives and preeclamptics respectively. The above findings seem to suggest that uncomplicated chronic hypertension in pregnancy is associated with normal birth weight which might be comparable to that of normal uncomplicated pregnancy. However, a recent study has demonstrated that chronic hypertension is associated with increased risk of preterm delivery, LBW and SGA infants and these adverse outcomes were more common in those managed on antihypertensives [21].

Moving forward, there is an urgent need to improve the quality of care provided to women with HDP in the country to optimize both maternal and perinatal outcomes associated these conditions. Frequent shortages of essential parenteral antihypertensive drugs such as hydralazine and labetalol as well as magnesium sulphate should be purposefully addressed. In Ghana, magnesium sulphate is first line drug for managing women with eclampsia and severe preeclampsia alongside antihypertensives. However, a recent nationwide survey indicated this essential drug was available in only 55% of health facilities providing maternity services at the time of data collection [22]. Other major challenges in the management of HDP in the country include poor laboratory support, delay in receiving care in health facilities, lack of adequately resourced neonatal intensive care unit (NICU) and poor referral system including transportation related issues. The biggest indisputable underlying factor associated with the above management related issues is extreme poverty characteristic of most developing countries like Ghana. We assert that the major perinatal adverse outcomes linked to HDP could be significantly improved if the above treatment-related challenges are minimized.

The strength of this study resides in the fact that we included women with singleton pregnancies complicated by all the various types of HDP displaying the relativity of the perinatal outcomes among the groups. This study serves as the baseline data on perinatal outcomes associated with HDP in the country upon which further studies could be referenced. The short duration of the study as well as the relatively small numbers involved are considered as limitations of the study. However, the findings from this study provides a broad idea about the perinatal health issues related to the management of HDP in Ghana and other West African countries with similar settings. Large longitudinal studies with high methodological quality and longer duration of data collection are recommended to better understand the dynamics of the perinatal outcomes associated with HDP and management strategies in Sub-Saharan Africa.

## Conclusion

Our study determined a significant burden of perinatal morbidity and mortality associated with HDP in the Ghanaian obstetric population and these adverse perinatal outcomes were more prevalent in preeclampsia compared to the other hypertensive disorders. We recommend a regular goal-oriented clinical audit into perinatal morbidity and mortality associated with HDP and a facelift of the defunct multidisciplinary approach to the management of these disorders in the hospital to improve the clinical outcomes of women with maternal hypertension.

## Abbreviations

APGAR: Appearance, Pulse, Grimace, Activity, and Respiration.
CI: Confidence Interval
HDP: Hypertensive Disorders in Pregnancy
IUGR: Intrauterine Growth Restriction
KBTH: Korle Bu Teaching Hospital
LBW: Low Birth Weight
NICU: Neonatal Intensive Care Unit
SGA: Small for Gestational Age
SSA: Sub-Saharan Africa
